# Epidemiological and Cytokine Profile of Patients with Pulmonary and Extrapulmonary Tuberculosis in a Population of the Brazilian Amazon

**DOI:** 10.3390/microorganisms10102075

**Published:** 2022-10-20

**Authors:** Maria Alice Freitas Queiroz, Sandra Souza Lima, Ednelza da Silva Graça Amoras, Francisca Dayse Martins de Sousa, Iury de Paula Souza, Juliana Abreu Lima Nunes, Igor Brasil-Costa, Izaura Maria Vieira Cayres-Vallinoto, Ricardo Ishak, Antonio Carlos Rosário Vallinoto

**Affiliations:** 1 Laboratory of Virology, Institute of Biological Sciences, Federal University of Pará (UFPA), Belem 66075-110, Brazil; 2 Graduate Program in Biology of Infectious and Parasitic Agents, Institute of Biological Sciences, Federal University of Pará (UFPA), Belem 66075-110, Brazil; 3 Laboratory of Immunology, Virology Section, Department of Health Surveillance, Evandro Chagas Institute, Ministry of Health, Ananindeua 67030-000, Brazil

**Keywords:** tuberculosis, extrapulmonary tuberculosis, immune response, epidemiology, cytokines

## Abstract

Several factors are associated with the development of different clinical forms of tuberculosis (TB). The present study evaluated epidemiological variables and cytokine levels in samples from 89 patients with TB (75 with pulmonary TB and 14 with extrapulmonary TB) and 45 controls. Cytokines were measured by flow cytometry (Human Th1/Th2/Th17 Cytometric Bead Array kit). The TB group had a higher frequency of individuals who were 39 years of age or older, married, with primary education or illiterate and had a lower family income (*p* < 0.05). All individuals with extrapulmonary TB reported that they were not working, and the main reasons were related to disease symptoms or treatment. The levels of IFN-γ (OR = 4.06) and IL-4 (OR = 2.62) were more likely to be elevated in the TB group (*p* = 0.05), and IFN-γ levels were lower in patients with extrapulmonary TB compared to those with pulmonary TB (OR = 0.11; *p* = 0.0050). The ROC curve was applied to investigate the diagnostic accuracy of IFN-γ levels between the different clinical forms of tuberculosis, resulting in high AUC (0.8661; *p* < 0.0001), sensitivity (93.85%) and specificity median (65.90%), suggesting that IFN-γ levels are useful to differentiate pulmonary TB from extrapulmonary TB. The dysregulation of pro- and anti-inflammatory cytokine levels represent a risk for the development of TB and contribute to the pathogenesis of the disease, especially variation in IFN-γ levels, which may determine protection or risk for extrapulmonary TB.

## 1. Introduction

Tuberculosis (TB) is caused by the bacterium *M. tuberculosis* and is the main infectious cause of death. Each year, approximately 10 million people become ill with TB. Despite being a preventable and curable disease, in 2019, there were 1.2 million deaths from TB among patients seronegative for HIV [1]. The progression of TB disease can be influenced by economic (low income), social (events that favour agglomerations) and individual (genetic or molecular changes in the host) factors [2,3,4].

Brazil is a member of the group of nations with a high TB burden, as established by the WHO, for having high estimates of incident cases of TB in the general population and among people living with HIV [1]. In 2019, 73,864 new cases of TB were reported in Brazil, corresponding to an incidence of 35 cases/100,000 inhabitants [5].

TB can be classified into different forms, the most common being pulmonary TB, when *M. tuberculosis* infection is restricted to the lungs [6,7]. Extrapulmonary TB is characterized by infection of almost all other body organs [8]. The risk of extrapulmonary TB increases with the progression of conditions that promote immunosuppression [9]. Approximately 10–50% of patients with extrapulmonary TB also have pulmonary involvement, but the disease can occur without the presence of pulmonary symptoms. The isolated extrapulmonary form represents 15–20% of all TB cases [10]. In addition, the bacterium can remain in the body without causing active infection and clinical manifestations, a form called latent TB infection (LTBI), which can be reactivated and cause disease [6,7].

The immune response elicited in the pulmonary microenvironment against the bacterium influences the regulation of the levels of inflammation acting to contain the infection [11]. Individuals infected with *M. tuberculosis* may not be able to contain the infection, despite the stimulation of cellular immunity, in the early progression to primary TB [12].

Infection by *M. tuberculosis* involves several cell groups characterized by different effector functions and phenotypic surface markers [13]. In this context, CD4+ T cells mediate immune protection, contributing to the establishment of inflammation and regulation of the immune response; among the prevalent groups of effector CD4+ T cells, the Th1 profile is associated with a large part of this protective response function [14,15]. The Th1 immune response profile is responsible for the recruitment of monocytes and granulocytes undertaken mainly by the cytokine IFN-γ, which induce the activation of antimicrobial activity in macrophages [14,16,17].

Several cytokines play an important role in the evolution of *M. tuberculosis* infection. Polyfunctional cells producing IFN-γ, TNF-α and IL2 are considered more appropriate for infection control, as they promote a more adequate activation of the cell’s effector functions against the bacillus [18]. Defects in the production of these cytokines, mainly IFN, are considered risk factors for *M. tuberculosis* infection and tuberculosis progression [19]. Reduced activity of this response type may favour the persistence of infection in the body. *M. tuberculosis* can manipulate the host’s immune system, modulating the processing of antigens to enable its replication, propagation and persistence, causing an imbalance in effector immune responses, mainly related to the production of cytokines [20,21,22].

Due to the high number of cases of TB, which may limit the lives of patients, and the development of extrapulmonary forms, which are difficult to diagnose, the present study evaluated the epidemiological characteristics of patients with pulmonary TB and extrapulmonary TB and compared the levels of the IFN-γ, TNF-α, IL-6, IL-2, IL-4, IL-10 and IL-17 cytokines between the groups of patients with different forms of TB and a control group to identify a concentration threshold for cytokines that would allow the molecular characterization of each type of TB.

## 2. Materials and Methods

### 2.1. Sample Characteristics and Collection

The study performed was observational cross-sectional and included 89 patients diagnosed with TB, i.e., 75 with pulmonary TB and 14 with extrapulmonary TB, including ocular (*n* = 1), pleural (*n* = 8), ganglionic (*n* = 2), miliary (*n* = 1) and meningo-encephalic (*n* = 2) TB, and a comparison group formed by 45 individuals who had contact with people with TB but who never developed the disease, referred to as the contact control group (CG). Individuals in the control group were negative for tuberculosis (TB) disease and self-reported having had contact with TB patients (family members or a close person). All subjects in the control group underwent a tuberculin skin test (TST), had a negative smear and a normal chest X-ray.

The diagnosis of tuberculosis cases was performed as established by the Brazilian Ministry of Health [23]. In addition to the clinical diagnosis, complementary tests were performed to confirm the diagnosis of tuberculosis. Cases of pulmonary TB were confirmed by smear microscopy of sputum or bronchial lavage fluid, followed by specific culture for *M. tuberculosis*.

Confirmation of extrapulmonary TB was made on different biological materials, including: lymph node aspirate or biopsy (ganglionic TB); pleural fluid for classification between exudate, with dosage of the enzyme adenosine deaminase (pleural TB); cerebrospinal fluid with cytometry, cytology and biochemistry (meningo-encephalic TB); and ocular fluid (ocular TB). All cases were confirmed by specific culture for *M. tuberculosis*. For miliary TB, radiography and computed tomography of the chest and suspicious areas were performed, which showed a diffuse micronodular pattern typical of this form of the disease.

The study was carried out from November 2020 to August 2021. The patients with TB were from João de Barros Barreto University Hospital (Hospital Universitário João de Barros Barreto-HUJBB) and from the outpatient clinic of the Municipal Health Unit of Guamá (Department of Municipal Health and Environment), both located in the city of Belém, the capital of the state of Pará, located in the North region of Brazil, which is part of the Brazilian Amazon region. The inclusion criteria were patients 18 years of age or older with a confirmed clinical and laboratory diagnosis of TB and without coinfection with HIV-1. Patients with diagnosed autoimmune disease were excluded. All patients were being treated for tuberculosis for a period ranging from 1 to 2 months.

Whole blood samples (10 mL) were collected using a vacuum collection system into tubes containing ethylenediaminetetraacetic acid (EDTA) as an anticoagulant and subsequently sent to the Laboratory of Virology, Institute of Biological Sciences of Federal University of Pará (LabVir-ICB/UFPA), where they were processed; the plasma samples were stored at −80 °C until use.

### 2.2. Plasma Cytokine Measurement

Cytokine levels were quantified by flow cytometry using a Human Th1/Th2/Th17 Cytometric Bead Array (CBA) kit (BD Biosciences, San Diego, CA, USA) in a BD FACS Canto II instrument. All procedures followed the manufacturer’s instructions; the method is based on beads conjugated to a capture antibody, i.e., six populations of beads with different fluorescence intensities, conjugated to a capture antibody specific for each cytokine, mixed to form the CBA and read in channel FL-3 in the flow cytometer. The bead populations were recorded on the basis of their respective fluorescence intensities, from the least bright to the brightest (IL-17 < IFN-γ < TNF-α < IL-10 < IL-6 < IL-4 < IL-2).

### 2.3. Tuberculin Skin Test (TST)

Control subjects submitted to a tuberculin skin test, performed by applying an intradermal injection of 0.1 mL (0.04 mcg) of PPD RT-23 (Mantoux, 2 UT/0.1 mL) in the middle third of the anterior surface of the left forearm, at an angle of 5 to 15 degrees, until the formation of a papule. The reading was performed 48 to 72 h after application, using a specific millimeter ruler, measuring the largest transverse diameter of the induration perpendicularly to the forearm. Results with induration greater than or equal to 5 mm were considered positive PPD [24].

### 2.4. Statistical Analysis

The data obtained were entered into a database in Microsoft Office Excel 2013 (Microsoft, Redmond, WA, USA). To describe the sociodemographic profile, descriptive statistics were used, categorical variables were presented as frequencies and percentages and the differences between the groups were evaluated using Fisher’s exact test and the G test. Normality analysis of the distribution of cytokine levels was performed using the Shapiro–Wilk test. Based on the results of the normality test, variations in the plasma levels of the markers between the groups were evaluated using the nonparametric Mann–Whitney test and simple and multiple logistic regression. Violin charts were used to show the dispersion of cytokine levels between the groups investigated. The Hosmer–Lemeshow test was used to evaluate the logistic regression model in relation to the observed and predicted values of the dependent variable. A good model fit is indicated by *p* > 0.05. Receiver operating characteristic (ROC) curves were created to investigate the diagnostic accuracy of IFN-γ levels between the different clinical forms of TB in relation to sensitivity and specificity. The area under the ROC curve (AUC) represented the ability of the model to correctly predict the participants with pulmonary TB and extrapulmonary TB forms of the disease. All tests were performed using Minitab 14.0 (Minitab, State College, PA, USA) and GraphPad Prism 5.0 (GraphPad Software, San Diego, CA, USA), and *p* values < 0.05 were considered significant.

## 3. Results

All selected individuals who presented positive TST (area with induration greater than or equal to 5 mm) were included in the control group.

The epidemiological characteristics of patients with TB and individuals in the CG are provided in Table 1. The TB group had a higher frequency of individuals who were 39 years of age or older (*p* = 0.0002), were married (*p* = 0.0212), had a primary education and were illiterate (*p* < 0.0001), had a lower family income (*p* < 0.0001) and were unvaccinated (BGC vaccine) or did not know their vaccination status.

The comparison between patients with pulmonary and extrapulmonary TB showed that most patients were young adults (18 to 38 years old) and that the pulmonary TB group had a higher frequency of individuals older than 59 years. Most patients with extrapulmonary TB had a low education level. The highest frequency of individuals with an income less than minimum wage was observed in the extrapulmonary TB group. In the pulmonary TB group, most patients were not working (60%), while none of the patients (100%) with extrapulmonary TB were working (*p* = 0.0044). Of the individuals who were not working, 66.7% of the pulmonary TB group and 92.9% of the extrapulmonary TB group reported treatment/disease as the reason. In the pulmonary TB group, the majority of patients lived in a home with four to six people, and in the extrapulmonary TB group, the frequency of living with 1–3 other individuals was higher. Most patients in both groups were vaccinated against *M. tuberculosis* (Table 2).

**Figure 1:**
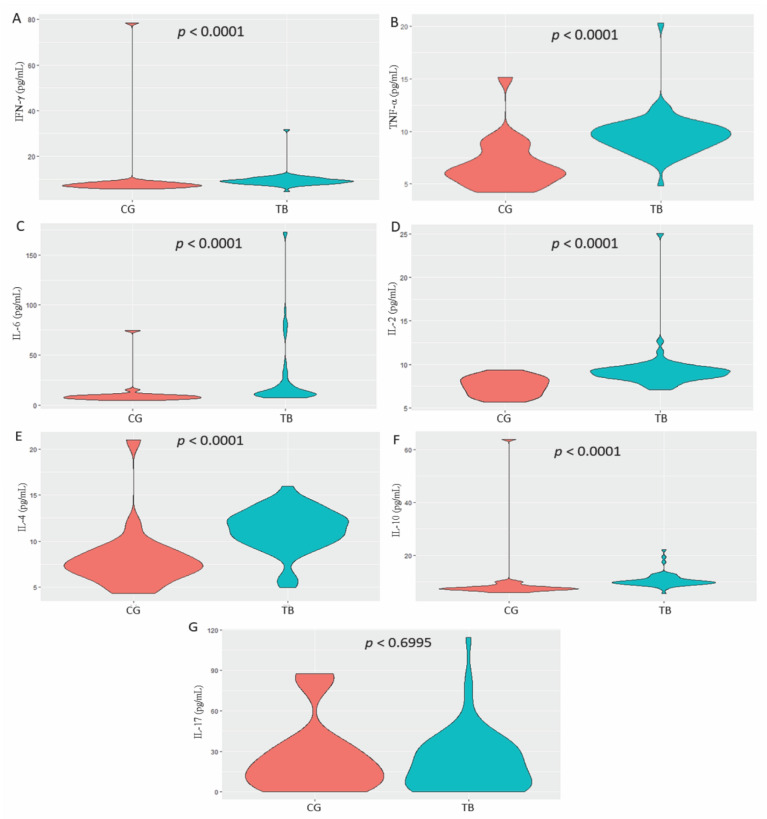
Comparison of: (**A**) IFN-γ; (**B**) TNF-α; (**C**) IL-6; (**D**) IL-2; (**E**) IL-4; (**F**) IL-10; and (**G**) IL-17 levels between the control group (CG) and tuberculosis (TB) group. Mann–Whitney test.

The comparison of serum cytokine levels indicated that the levels of IFN-γ, TNF-α, IL-6, IL-2, IL-4 and IL-10 were significantly higher in the TB group (*p* < 0.0001; Figure 1A–F); only IL-17 was not significantly different between the two groups (Figure 1G).

**Figure 2:**
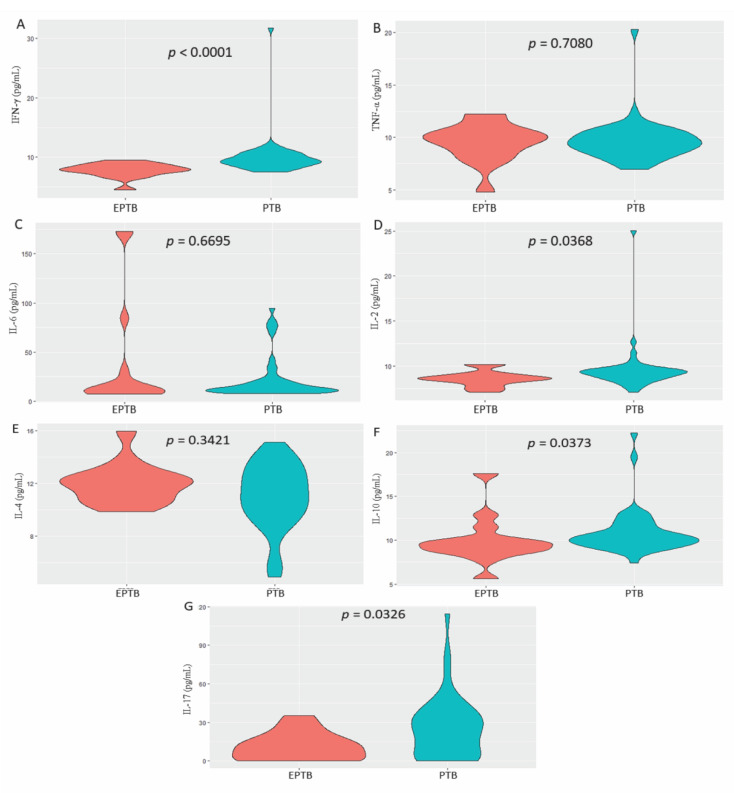
Comparison of: (**A**) IFN-γ; (**B**) TNF-α; (**C**) IL-6; (**D**) IL-2; (**E**) IL-4; (**F**) IL-10; and (**G**) IL-17 levels between the pulmonary tuberculosis (PTB) and extrapulmonary tuberculosis (EPTB) groups. Mann–Whitney test.

In the comparison of median cytokine levels between patients with pulmonary TB and extrapulmonary TB, the extrapulmonary TB group had significantly lower levels of IFN-γ (*p* < 0.0001; Figure 2A), IL-2 (*p* = 0.0368; Figure 2D), IL-10 (*p* = 0.0373; Figure 2F) and IL-17 (*p* = 0.0326; Figure 2G).

Comparison of cytokine levels among individuals with different age groups evaluated in the study showed that TB patients aged over 59 years had lower levels of all cytokines, with significant differences for IL-2, IL-4 and IL-10 (*p* < 0.05). In the control group, IFN-γ and TNF-α levels were significantly lower in older individuals (*p* < 0.05; Table 3).

Since the group with extrapulmonary TB had reduced levels of IFN-γ compared to the TB group and the majority were aged between 18 and 39 years, an assessment of IFN-γ levels was performed with only patients with pulmonary TB among the different age groups, which revealed a significant difference (*p* < 0.05) between the 18–38 age group (median = 10.14; IIQ = 1.72) and the >59 years group (median = 8.38; IIQ = 1.65).

The analysis of cytokine levels between the CG who were positive for TST and TB groups by simple logistic regression indicated that with the exception of IL-6 and IL-17, high levels of all other cytokines represented a risk for the development of active TB. However, when evaluating the interaction between cytokine levels through multiple logistic regression, only high levels of IFN-γ and IL-4 were significant risks for the disease (Table 4).

The comparison of cytokine levels between the pulmonary and extrapulmonary TB groups showed through simple and multiple logistic regression that IFN-γ levels were lower in patients with extrapulmonary TB compared to those with pulmonary TB (Table 5).

**Figure 3:**
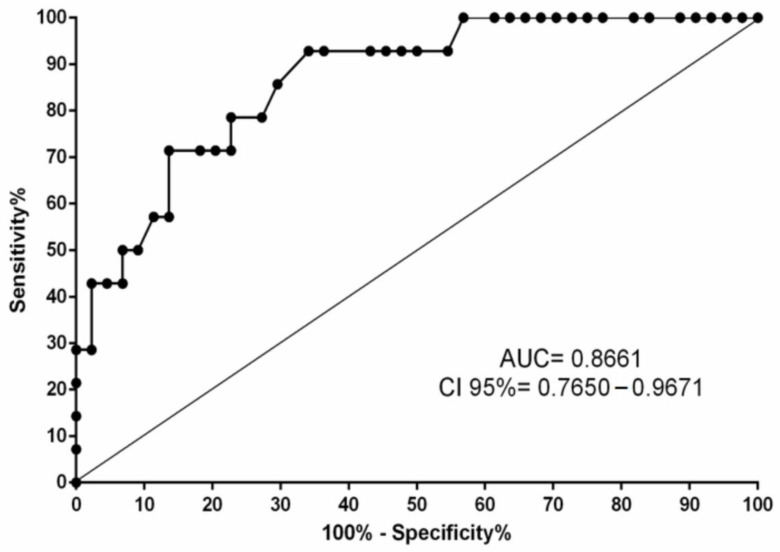
ROC curve for IFN-γ levels for the diagnostic prediction of extrapulmonary tuberculosis.

Using a regression model, IFN-γ levels were evaluated for the diagnostic prediction of extrapulmonary TB. A ROC curve was used to determine the best cut-off point (0.1478), which corresponded to an AUC of 0.8661 (*p* < 0.0001), sensitivity of 93.85% and specificity of 65.90% (Figure 3).

## 4. Discussion

Tuberculosis is a disease with a large global impact, occurring mainly in adults and characterized by being highly infectious [1]. Several factors are associated with susceptibility to *M. tuberculosis* and the development of different clinical forms of TB, including socioeconomic factors and factors related to the host immune response [2,3,25,26].

The comparison of socioeconomic characteristics between individuals with TB and the CG showed that the group with TB had a higher frequency of individuals who were older, were married, had a lower educational level, and had a lower income. The higher rate of TB in older adults may be related to the presence of LTBI in these patients and other comorbidities and may also be a reflection of the reduced immune response [27]. During aging, there is a reduction in the production of immune cells, especially T lymphocytes from the age of 50 [28]. This result shows that older individuals who had contact with the bacillus are more likely to develop the disease compared to younger individuals. Therefore, elderly patients need to be monitored to identify the disease at an earlier stage.

A low level of education promotes a lack of knowledge about the disease, especially about prevention and control measures. A low family income hinders access to an adequate diet and to health services, which are essential for controlling TB. The link between TB and poverty is well established, as poverty is associated with a longer duration of the disease because poorer people have a higher rate of effective contact (resulting from agglomerations and poor housing conditions), which contributes to maintenance of the disease [25]. Therefore, social public policies for combating poverty can also serve as public health policies as they contribute to the fight against various diseases, including TB.

In the analysis of socioeconomic variables between patients with pulmonary TB and those with a more complex form of the disease, extrapulmonary TB, there was an association of frequencies for the variable level of education, related to the consequences of the disease. Most patients with extrapulmonary TB had a lower level of education compared to patients with pulmonary TB, which reflects a lower knowledge about health–disease processes and lower financial resources. All individuals with extrapulmonary TB reported that they were not working, and the main reasons were related to disease symptoms or treatment. Although extrapulmonary TB is historically neglected due to its scarce epidemiological impact on disease transmission (the concern with transmission is not as important as that in patients with pulmonary TB), the difficulty of diagnosis promotes a delay in treatment, which can lead to increased disease severity and economic expenditures for patients and the affected families [29,30]. Extrapulmonary TB was also responsible for the interruption of work activities in approximately 83% of patients evaluated in a study conducted in South Africa [30].

Although the majority of tuberculosis cases are pulmonary TB, responsible for most hospitalizations for tuberculosis, extrapulmonary TB causes a significant number of hospitalizations, which have a longer hospitalization time and generate a higher cost compared to pulmonary TB, although there is not a difference in mortality rates. However, miliary TB and TB of the meninges and central nervous system had an increased risk of mortality [31]. These data show that extrapulmonary TB has a significant impact on the lives of those affected; however, there is little information about the social impact of the disease, which needs to be better investigated.

The serum levels of most of the evaluated cytokines were higher in the TB group than in the CG, showing that active infection by *M. tuberculosis* induces a highly reactive immune response, which can lead to the activation of different cell populations and the production of cytokines specific or not for the eradication of infection [32,33,34]. Among the evaluated cytokines, IFN-γ and TNF-α stand out for being fundamental in the control of *M. tuberculosis* infection, as they contribute to the recruitment and activation of innate immune cells, such as monocytes and granulocytes and activation of T lymphocytes cytotoxics [15,35]. The TB group had higher levels of these two cytokines compared to the control; however, patients with extrapulmonary TB had lower levels of IFN-γ compared to patients with pulmonary TB. This group of individuals appears to have a deficient immune response, mainly influencing the production of IFN-γ, which can impair the resolution of the infection.

In the assessment of cytokine levels in relation to different age groups, it was possible to observe a reduction in the levels of most cytokines evaluated in patients aged 60 years or older in the TB group; however, no significant difference was observed in the levels of IFN-γ. As mentioned above, the aging process promotes a reduction in the production of immune cells and consequently in the immune–inflammatory response [28]. However, the lack of significance in IFN-γ levels in relation to age may be due to the fact that most individuals with extrapulmonary TB were younger (aged 18–38) and had lower cytokine levels. In an age-related analysis of patients with pulmonary TB alone, IFN-γ levels were significantly lower in older patients.

The balance between the levels of various cytokines is essential for the resolution of *M. tuberculosis* infection or for the progression of TB. Higher levels of proinflammatory and anti-inflammatory cytokines in patients with TB were also observed in other studies [36,37,38]. In this context, the levels of anti-inflammatory cytokines increase with the body’s attempt to prevent intense damage caused by the action of proinflammatory cytokines, which are produced to fight the bacterium. However, evidence suggests that the activation of the Th2 response within a proinflammatory environment leads to bacterial proliferation and disease progression [39,40].

The presence of *M. tuberculosis* infection in an environment with a predominance of anti-inflammatory cytokines possibly results in difficulty in controlling the infection and spread of the bacillus [18]. The multiple regression analysis results showed that the elevated levels of IFN-γ and IL-4 represented a 4- and 2.5-fold risk for the development of active TB, respectively, which is probably related to the fact that elevated cytokine levels also influence the pathogenesis of the disease. Thus, the high IFN-γ and IL-4 levels in patients with TB suggest an attempt by the body to balance the Th1-Th2 responses. However, an effective response against *M. tuberculosis* is mediated by the activation of the Th1 pathway, and high IL-4 levels will favour infection. IL-4 can subvert mycobacterial containment in human macrophages, probably through disturbances in the pathways linked to Treg and Th1 cells [41].

For an effective response against *M. tuberculosis*, during cell differentiation induced by exposure to mycobacterial antigens, CD4+ or CD8+ T cells must acquire the ability to produce various cytokines, especially IFN-γ, TNF-α and IL-2 [37]. These cytokines are extremely necessary to activate the Th1 pathway, induce IFN-γ and consequently activate macrophages *and M. tuberculosis* death/restriction. Mice deficient in the CD4+ T cell subset and Th1 cytokines were associated with a rapid progression to infection-related death [14,42].

Regarding extrapulmonary TB, lower levels of IFN-γ, IL-2, IL-10 and IL-17 were observed in patients in this group compared with pulmonary TB. The spread of the bacillus to other sites in the body outside the lung is still not well understood, but it is believed that factors related to the bacterium and the host contribute to the establishment of extrapulmonary TB [43]. Extrapulmonary TB has been shown to be associated with immune system deficiency, mainly related to decreased Th1 cell activation and IFN-γ secretion in response to mycobacterial antigens [44]. The results of the present study show a reduced immune–inflammatory response related to extrapulmonary TB, which was also observed in other studies [45,46]. Additionally, based on the evaluation of cytokine levels by simple and multiple linear regression, the main “molecular signature” of this immune deficiency is associated with low IFN-γ levels. The results of multiple logistic regression suggest that for each addition of 1 pg/mL of IFN-γ, the chance of an individual developing extrapulmonary TB is reduced by nine times. The significant differences in the levels of this cytokine allowed us to evaluate its efficiency in differentiating between pulmonary and extrapulmonary TB. Using a ROC curve, it was determined that this cytokine is a good marker for differentiating the forms of TB, with 93.85% sensitivity and 65.90% specificity. Thus, the measurement of IFN-γ could function as a screening aid in the diagnosis of suspected extrapulmonary TB in HIV-seronegative patients.

The study’s limitation consisted of the reduced sample number of extrapulmonary TB. As the focus of the study was to assess factors related to the disease caused specifically by *M. tuberculosis*, many cases were excluded because they were co-infected with HIV.

## 5. Conclusions

In summary, extrapulmonary TB has a significant socioeconomic impact on the lives of individuals with the disease, making it impossible for them to work. Increased levels of pro- and anti-inflammatory cytokines, which are related to the containment of infection, but also contribute to the pathogenesis of the disease, represent risk factors for TB development. In contrast, the risk of extrapulmonary TB was associated with low levels of the proinflammatory cytokine IFN-γ compared to pulmonary TB, indicating that the disease results from the weak activation of the immune response, mainly related to the Th1 response.

## Figures and Tables

**Table 1:** Epidemiological characterization of patients with TB and individuals in a control group.

Characteristic	Tuberculosis Group *n* = 89 *n* (%)	Control Group *n* = 45 *n* (%)	*p*
**Age**			
18–38	39 (43.8)	35 (77.8)	0.0002 *
39–59	33 (37.1)	9 (20.0)	
>59	17 (19.1)	1 (2.2)	
**Sex**			
Female	43 (48.3)	25 (55.6)	0.5426 **
Male	46 (51.7)	20 (44.4)	
**Marital status**			
Married	32 (36.0)	8 (17.8)	0.0212 *
Single	43 (48.3)	33 (73.3)	
Separated/divorced/widowed	14 (15.7)	4 (8.9)	
**Education level**			
Primary education/Illiterate	40 (44.9)	2 (4.4)	<0.0001 *
Secondary education	39 (43.8)	24 (53.4)	
Higher education	10 (11.3)	19 (42.2)	
**Family income (minimum wage) ^a^**			
<1	17 (19.1)	1 (2.2)	<0.0001 *
1–3	65 (73.0)	24 (53.4)	
4–6	7 (7.9)	15 (33.3)	
>10	0	5 (11.1)	
**Number of people living in the home**			
1–3	31 (31.4)	23 (51.1)	0.2009 *
4–6	49 (60.0)	19 (42.2)	
≥7	9 (8.6)	3 (6.7)	
**Have you been vaccinated against TB?**			
Yes	75 (84.3)	45 (100)	0.1106 *
No	6 (6.7)	0	
Does not know	8 (9.0)	0	

*n* = number of individuals; ^a^ USD 225.70; * G test; ** Fisher’s exact test.

**Table 2:** Epidemiological characterization of patients with pulmonary TB and extrapulmonary TB.

Characteristic	Pulmonary TB *n* = 75 *n* (%)	Extrapulmonary TB *n* = 14 *n* (%)	*p*
**Age**			
18–38	31 (41.3)	8 (57.2)	0.3526 *
39–59	28 (37.4)	5 (35.7)	
>59	16 (21.3)	1 (7.1)	
**Sex**			
Female	35 (46.7)	8 (50.0)	0.5656 **
Male	40 (53.3)	6 (50.0)	
**Marital status**			
Married	28 (37.3)	4 (28.6)	0.1179 *
Single	36 (48.0)	7 (50.0)	
Separated/divorced/widowed	11 (14.7)	3 (21.4)	
**Education level**			
Primary education/Illiterate	30 (40.0)	10 (71.4)	0.0449 *
Secondary education	35 (46.7)	4 (28.6)	
Higher education	10 (13.3)	0 (0.0)	
**Family income (minimum wage) ^a^**			
< 1	13 (17.8)	4 (28.6)	0.2484 *
1–3	55 (73.3)	10 (71.4)	
4–6	7 (8.9)	0 (0.0)	
**Are you working?**			
Yes	33 (44.0)	0 (0.0)	0.0008 **
No	42 (55.0)	14 (100)	
**Reason for not working**			
Treatment/disease	28 (66.7)	13 (92.9)	0.0822 **
Other	14 (33.3)	1 (7.1)	
**Number of people living in the home**			
1–3	24 (31.4)	7 (50.0)	0.0917 *
4–6	45 (60.0)	4 (28.6)	
≥7	6 (8.6)	3 (21.4)	
**Have you been vaccinated against TB?**			
Yes	65 (86.6)	10 (71.5)	0.3307 *
No	5 (6.7)	1 (7.1)	
Does not know	5 (6.7)	3 (21.4)	

*n* = number of individuals; ^a^ USD 225.70; * G test; ** Fisher’s exact test.

**Table 3:** Comparison of cytokine levels according to the age group of patients with TB and individuals in the control group.

Cytokines/Ages	TB Median (IIQ)	*p* ^1^	CG Median (IIQ)	*p* ^2^
**IFN-γ**				
18–38	9.36 (2.07)	0.1229	8.01 (1.66)	0.0706
39–59	9.23 (2.08)		7.27 (0.90)	
>59	7.98 (2.00)		6.62 (0.00) *	
**TNF-α**				
18–38	9.96 (1.46)	0.1317	6.71 (1.92)	0.0294
39–59	9.34 (1.72)		5.85 (1.76)	
>59	8.82 (2.68)		4.41 (0.00) *	
**IL-6**				
18–38	12.21 (29.19)	0.4948	7.01 (1.82)	0.1023
39–59	13.10 (11.03)		7.97 (1.41)	
>59	11.03 (4.59)		6.89 (0.00) *	
**IL-2**				
18–38	9.05 (1.26)	0.0344	7.33 (1.39)	0.4127
39–59	9.25 (0.88)		7.44 (1.74)	
>59	8.23 (1.15)		6.01 (0.00) *	
**IL-4**				
18–38	12.29 (3.71)	0.0355	6.85 (1.04)	0.0333
39–59	11.17 (2.48)		7.74 (1.81)	
>59	10.66 (3.84)		7.13 (0.00) *	
**IL-10**				
18–38	10.02 (2.66)	0.0106	7.53 (0.82)	0.5973
39–59	10.27 (1.74)		7.31 (1.14)	
>59	9.48 (1.15)		7.12 (0.00) *	
**IL-17**				
18–38	21.77 (26.34)	0.4272	17.14 (16.67)	0.9605
39–59	24.63 (34.92)		16.65 (10.28)	
>59	15.32 (20.68)		16.11 (0.00) *	

* sample number equal to 1; *p*^1^: Kruskal–Wallis test; *p*^2^: Mann–Whitney test.

**Table 4:** Evaluation of cytokine levels by logistic regression between the control and TB groups.

Cytokine	Simple Logistic Regression			Multiple Logistic Regression *		
	OR	95% CI	*p*	OR	95% CI	*p*
IFN-γ	2.98	1.69–5.28	0.0002	4.06	1.79–9.21	0.0008
TNF-α	2.28	1.55–3.34	<0.0001	-	-	-
IL-6	1.06	0.98–1.13	0.1383	-	-	-
IL-2	5.02	2.34–10.72	<0.0001	-	-	-
IL-4	1.61	1.27–2.06	0.0001	2.62	1.58–4.33	0.0002
IL-10	4.13	2.15–7.94	<0.0001	-	-	-
IL-17	0.99	0.97–1.01	0.3349	-	-	-

* Hosmer–Lemeshow chi-square = 4.2738; degrees of freedom = 8; *p* = 0.8320.

**Table 5:** Evaluation of cytokine levels by logistic regression between the group with pulmonary TB and extrapulmonary TB.

Cytokine	Simple Logistic Regression			Multiple Logistic Regression *		
	OR	95% CI	*p*	OR	95% CI	*p*
IFN-γ	0.1994	0.0752–0.5286	0.0012	0.1064	0.0223–0.5080	0.0050
TNF-α	0.9613	0.6937–1.3321	0.8125	-	-	-
IL-6	1.0146	0.9975–1.0319	0.0942	-	-	-
IL-2	0.4834	0.2239–1.0437	0.0642	-	-	-
IL-4	1.2302	0.907–1.6685	0.1827	-	-	-
IL-10	0.8233	0.5861–1.1566	0.2623	-	-	-
IL-17	0.954	0.9123–0.9976	0.0388	-	-	-

* Hosmer–Lemeshow chi-square = 3.2605; degrees of freedom = 8; *p* = 0.9170.

## Data Availability

The data analyzed in this study are included within the paper.
